# Hypolipidemic and Antioxidant Effects of *Guishe* Extract from *Agave lechuguilla,* a Mexican Plant with Biotechnological Potential, on Streptozotocin-Induced Diabetic Male Rats

**DOI:** 10.3390/plants10112492

**Published:** 2021-11-18

**Authors:** Edgar R. Esquivel-Gutiérrez, Salvador Manzo-Avalos, Donovan J. Peña-Montes, Alfredo Saavedra-Molina, Zoé P. Morreeuw, Ana G. Reyes

**Affiliations:** 1 Centro de Investigaciones Biológicas del Noroeste S.C., Av. Instituto Politécnico Nacional 195, Col. Playa Palo de Santa Rita Sur, La Paz C.P. 23096, Baja California Sur, Mexico; edgar.esquivel@umich.mx (E.R.E.-G.); zpelletier@pg.cibnor.mx (Z.P.M.); 2 Instituto de Investigaciones, Químico Biológicas, Universidad Michoacana de San Nicolás de Hidalgo, Morelia C.P. 58030, Michoacan, Mexico; smanzo@umich.mx (S.M.-A.); 0618853j@umich.mx (D.J.P.-M.); saavedra@umich.mx (A.S.-M.); 3 CONACYT—Centro de Investigaciones Biológicas del Noroeste S.C., Av. Instituto Politécnico Nacional 195, Col. Playa Palo de Santa Rita Sur, La Paz C.P. 23096, Baja California Sur, Mexico

**Keywords:** diabetes, dyslipidemias, flavonoids, oxidative stress, saponins

## Abstract

In the present study, we used a by-product from *Agave lechuguilla* (*guishe*) to test its antidiabetic effect, hypolipidemic activity, and capacity to mitigate the oxidative stress in kidney mitochondria from streptozotocin-induced diabetic rats. Orally, a crude aqueous extract from lyophilized *guishe* was administered over 5 weeks at different doses. Blood glucose and body weight were monitored. Also, blood chemistry, bilirubin, and alanine aminotransferase were assayed. Furthermore, the activity of catalase, thiobarbituric acid reactive species, mitochondrial superoxide dismutase, glutathione and glutathione peroxidase were determined in isolated kidney mitochondria. Our results show that *guishe* extracts have no antidiabetic properties at any dose. Nevertheless, it was able to diminish serum triglyceride levels and regulate the oxidative stress observed in isolated kidney mitochondria. These observations indicate that the aqueous extract from *guishe* can be used to treat abnormalities in serum lipids, as a hypolipidemic, and mitigate the oxidative stress, as an antioxidant, occurring during diabetes.

## 1. Introduction

Diabetes mellitus (DM) is a long-lasting health condition that affects glucose metabolism. Over time, it is also a strong and common risk factor for chronic kidney disease, nephropathy being the most common cause for end-stage renal disease [1]. In 2019, the number of people with DM increased globally by over two times more than during the last three decades. In Mexico, it has been growing steadily at approximately 25% every six years since 2000 [2]; for this reason, DM remains the most important public health problem worldwide [3]. Historically, the diagnosis of DM was presumptively made based on symptomatology. Nonetheless, in the current times, a simple blood test can effectively diagnose this pathology [4].

Diabetic patients have an increased risk of developing cardiovascular diseases, due to the altered lipid metabolism. Abnormal serum lipids are frequently present before diabetes onset; thus, dyslipidemia (high triglycerides and cholesterol) becomes a cardiovascular risk factor for DM and its complications. Due to the latter, lipid control and careful monitoring are typically recommended [5]. Notwithstanding, not only do hyperglycemia and lipid alterations affect health conditions, but increased oxidative stress also causes impairment in physiological functions [6]. Oxidative stress could be mitigated by the main antioxidant enzymes in biological systems, including superoxide dismutase (SOD), catalase (CAT), and glutathione peroxidase (GPx). Glutathione (GSH/GSSG) is another antioxidant that has been extensively studied because of its interaction with glutathione-dependent antioxidant enzymes [7]. To reduce oxidative stress, many medicinal plants exist, with antioxidant benefits for diabetes and its complications [8].

Extracts from herbal medicines have been prepared and used, and their usefulness has been evaluated in experimental DM models in animals. In some plants, active hypoglycemic principles have been isolated and their mechanism of action studied, and most of them could be more effective due to their high antioxidant activity [6]. *Agave lechuguilla* Torrey (Asparagaceae) has been used in DM treatment [9]; however, the authors did not provide further information beyond mentioning its hypoglycemic effect without scientific evidence. Agave species were probably second only to maize (corn) in the development of agriculture in Mesoamerica. Nowadays, their juice is used industrially for beverages and food additives [10]. Particularly, *Agave lechuguilla* is mainly distributed in the northeast of Mexico and is mainly used to extract fibers, resulting in 15% fibers and 85% of a by-product waste named *guishe* [11]. *Guishe* is a bagasse left after fiber extraction; it is a vegetal pulp without any use. Normally, the producers discard this pulp to open-field, causing environmental problems; therefore, some Mexican groups have proposed a biorefinery model as an alternative to overcome this problem [12]. In Mexico, the valorization of *guishe* has become a national priority. To this end, Morreeuw et al., 2021 [13,14], showed that the crude extracts of lechuguilla contain phytochemicals with beneficial effects against DM and some other diseases, specifically, some anthocyanins, such as cyanidin and hesperidin. Both kinds of molecules have shown antidiabetic and hypolipidemic effects by inducing phosphorylation of the insulin receptor in liver and increased glucose uptake in primary adipocytes, respectively, in diabetic rats [15,16]. Additionally, it has been shown that the aqueous extracts of *guishe* contain an important fraction of saponins [12]. Saponins are known to have antidiabetic properties [17]. Thus, as part of a biotechnological strategy to give value to this important by-product, a crude aqueous extract from *guishe* was used in the present study, and the main aim was to evaluate its effects in streptozotocin-induced diabetic rats (STZ-DM) on blood glucose (BG) levels, serum lipid profile, and kidney mitochondria oxidative stress.

## 2. Results and Discussion

We investigated the effects on DM of an aqueous extract of a by-product from *A. lechuguilla* called *guishe*. Different doses (150, 300, and 600 mg/kg) were tested, with the main objective to elucidate whether the extract possessed properties to reduce blood glucose in STZ-DM rats. Prior to DM induction, all animals were normoglycemic. Once DM was expressed, blood glucose was registered every 5 days, and results showed that the administration of *guishe* over 5 weeks had no hypoglycemic effects at any doses tested. At the end of the treatments, serum glucose concentrations were significantly higher in all diabetic groups (*p* < 0.05) compared to controls, which means that the aqueous extract of *guishe* has no hypoglycemic effects (Table 1). Increased levels of blood glucose were caused by the toxic effects of STZ, which can destroy pancreatic β cells, and not due to the aqueous extract of *guishe*; all control animals (treated or not) were normoglycemic (Table 1). DM was established at a fasting blood glucose level >200 mg/dL. Our results show that the aqueous extract of *guishe* had no effects in reducing hyperglycemia in DM rats, nor did it modify blood glucose levels in control animals at any dose. Reports [9] with no scientific evidence or further information mentioned that *A. lechuguilla* had been used as a DM treatment. At the least, *guishe* as a by-product does not possess antidiabetic properties.

Hyperlipidemia is a complication associated with diabetes mellitus due to abnormalities in lipoproteins. Pharmacologic and herbal medicines are capable of controlling blood sugar levels as well as preventing heart and vascular disease, which are frequent complications of diabetes [8]. Our results showed that the administration of *guishe* extract significantly improves triglyceride levels. The administration of *guishe* over 5 weeks, at a dose of 300 mg/kg, significantly decreased the levels of serum triglycerides in DM rats, whereas a dose of 150 or 600 mg/kg did not produce a significant reduction in serum triglyceride levels. However, an important observation was that control animals responded to the *guishe* treatment, starting at a dose of 150 mg/kg, with a significant reduction (*p* < 0.05) in this important parameter in healthy animals (Table 1). Studies have shown that elevated total or low-density lipoprotein (LDL) cholesterol levels in the blood are powerful risk factors for coronary heart disease. *Guishe* extract did not alter the total serum cholesterol levels in any treated group.

Hyperglycemia, the main feature in diabetes, contributes to the development of diabetic nephropathy; it can be evaluated by measuring serum creatinine [1]. In this study, when compared to baseline creatinine values, none of the groups presented statistically different serum creatinine (0.4–1.4 mg/dL) levels at the end of the treatments. Five weeks with DM is not enough to develop diabetic nephropathy, but, importantly, *guishe* did not affect the renal function, because it also did not modify the uric acid levels (0.97–4.72 mg/dL). A comparison of the baseline urea levels with values obtained after 5 weeks showed that there were significant differences (*p*  <  0.05). Normal values for urea range from 39.6 to 68.8 mg/dL [18]; control rats presented normal values, even those control animals that received 150 mg/kg of extract. Diabetes causes an increase in serum urea (Table 1); however, an increase was observed even in treated DM rats (*p* < 0.05) at any dose tested. The same increase was observed in controls treated with 300 or 600 mg/kg of *guishe* extract. In consequence, *guishe* extracts had no effects on urea levels; however, urea is not generally considered to be an important uremic toxin—despite elevation of this nitrogenous waste product—except at very high concentrations [19].

We can also observe that *guishe* does not affect liver function, because no effect on total bilirubin (0.0–0.64 mg/dL) was observed. Moreover, baseline levels of ALT are summarized in Table 1. When compared with baseline values, no significant increase of ALT (52–224 U/L) levels was observed in control animals. However, a significant increase (*p* < 0.05) in the level of this hepatic marker enzyme was seen in treated DM but not in those that received the vehicle (Table 1). ALT is a hepatic enzyme that helps to convert proteins into energy when the cells cannot use glucose as an energy source in DM. Thus, ALT is released into the bloodstream, increasing its normal values [20]. Further studies must be performed to validate these results. AST (aspartate aminotransferase) is a marker that can explain ALT increases, but it is unspecific in the rat liver; therefore, it was not determined [18]. Normally, increased values for ALT are associated with metabolic disorders, such as hyperlipidemia and obesity, and including diabetes—specifically, hepatic insulin resistance sensitivity, which is a risk factor of type 2 diabetes [20,21,22]. In any case, different doses must be tested to validate the beneficial effect of the aqueous extract.

The kidney is considered to have the greatest number of mitochondria per tissue mass; however, in the presence of hyperglycemia from type 1 DM, the kidney response has been found to reduce the content of mitochondria per tissue mass. Additionally, it has been widely considered that overproduction of superoxide (the main reactive oxygen species (ROS)) from mitochondria plays a key role in causing diabetic complications and diabetic kidney disease [23]. For this reason, our research team is interested in elucidating the behavior of kidney mitochondria when a DM treatment is applied, especially those treatments based on medicinal plants.

To evaluate the effects of *guishe* on the redox status in DM, we studied parameters of oxidative stress in control and STZ-DM rats. Diabetic nephropathy (DN) develops in diabetic patients. The mitochondrial electron transport chain produces most of the ROS that are involved in DM [24]. To determine the effects of *guishe* on the oxidative stress caused by DM, we first evaluated lipid peroxidation by means of TBARS in kidney mitochondria from each group. No significant differences were found among all experimental groups (data not shown). Hyperglycemia causes glucose auto-oxidation, protein glycation, and activation of polyol metabolic pathways, which further accelerates the formation of ROS. The formation of ROS can increase lipid modifications in various tissues, including the kidney during DM [23].

The role of oxidative stress in the pathogenesis of DM and its complications is well established. In the present study, to evaluate the antioxidant effect of the *guishe* extract, we assayed lipid peroxidation. Administration of the extract (150, 300, or 600 mg/kg) for 5 weeks was able to prevent lipid peroxidation. We also assayed the nitric oxide values, obtained with the Griess reagent and nitrosylation profile by western blot (anti 3-nitrotyrosine), in isolated kidney mitochondria, and no significant differences were observed between the groups (data not shown). Crude extracts from lyophilized *guishe* were analyzed by Peña-Rodriguez et al., 2020 [11], identifying numerous bioactive phytochemicals, such as saponins and flavonoids. We suggest that they are responsible, alone or in combination, for various pharmacological activities, including antioxidant and hypolipidemic, because their chemical analysis revealed the presence of chlorogenin, diosgenin, diosgenin diglucoside, esmilagenin, hecogenin, manogenin, tigogenin hexose, yucagenin, and flavonols, such as quercetin.

**Figure 1:**
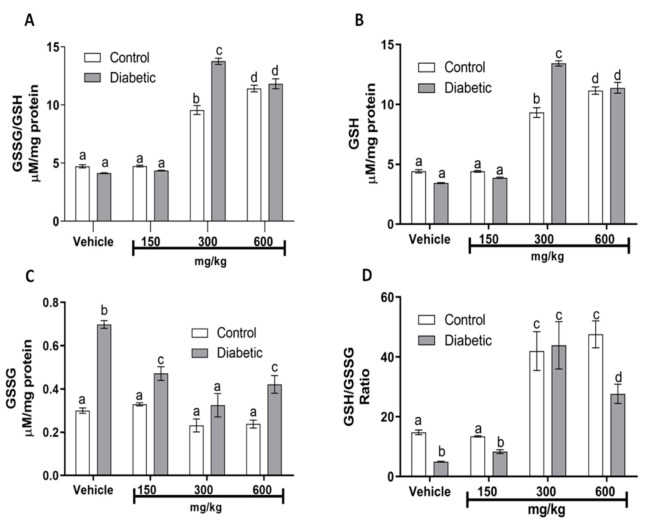
Effects of *guishe* on redox biomarkers (glutathione) in kidney mitochondria. (**A**) Total glutathione (GSSG/GSH); (**B**) reduced glutathione (GSH); (**C**) oxidized glutathione (GSSG); (**D**) glutathione ratio (GSH/GSSG ratio). GSSG/GSH and GSSG were determined in pools from isolated kidney mitochondria from each group—in all cases, at the end of the 5 weeks of treatments. Values express the mean ± SEM of at least *n* = 3 rats. ANOVA with post hoc Tukey HSD test for unbalanced data was performed. Means not sharing the same superscript are significantly different (*p* ≤ 0.05). Source: authors’ own elaboration.

DM results in an imbalance between protective antioxidant enzymes and increased production of free radicals [24]. One such antioxidant is the endogenous enzyme glutathione peroxidase (GSH-Px); this is a major pathway of H_2_O_2_ metabolism and catalyzes the reduction of other peroxides. It is, thus, important for the protection of membrane lipids against oxidation. Table 2 shows the differences in GSH-Px enzyme levels between the control and DM groups, which were significant (*p* < 0.05). The decrease in GSH-Px levels, compared to the control group, is due to the oxidative stress that occurs in DM, suggesting that the pathology affects the enzyme activity. Even at the 150 mg/kg dose, the extract did not confer any protection, although it enhanced the GSH-Px activity in both groups. Surprisingly, in both groups, the largest increase in GSH-Px activity was observed at the doses of 300 and 600 mg/kg of the *guishe* extract, confirming the beneficial effect on the redox balance. As was expected, no significant changes were observed in catalase activity (Table 2) when assayed in kidney homogenates, because it correlates with GSH-Px activity. Other important antioxidant molecules that contain thiol groups are glutathione species (GSH/GSSG). Several human diseases are associated with deficiencies of specific enzymes of GSH metabolism. Our glutathione analysis in kidney mitochondria showed an important redox control, because total glutathione (Figure 1A) was significantly increased (*p* < 0.05) in the DM group that received 300 mg/kg of *guishe* extract, most of it corresponding to GSH (Figure 1B). As observed, oxidized glutathione (GSSG) is increased significantly in DM groups that received the vehicle, 150 or 600 mg/kg, compared with their corresponding control group (Figure 1C). The GSH/GSSG ratio (Figure 1D) indicates that 300 mg/kg was the best dose to ameliorate the DM alterations in redox balance.

On the other hand, with the main purpose of evaluating other antioxidant enzymes such as the mitochondrial SOD (MnSOD), we investigated MnSOD activity. The *guishe* extract increased MnSOD activity and conferred protection against oxidants produced in this organelle. Table 2 shows a significant (*p* < 0.05) increase of MnSOD activity in the DM group. At a dose of 150 mg/kg, the extract did not confer significant protection against the redox imbalance due to DM. However, at the dose of 300 mg/kg, it not only protected but rather increased the activity of MnSOD. 

Our results showed that treatment with an aqueous extract of *guishe* reduced serum triglyceride levels and mitigated the oxidative stress, suggesting that these effects were mediated by the saponins and flavonoids, especially quercetin, present in the extract because of their direct radical scavenging action, antioxidative action, inducible nitric oxide synthesis inhibitory action, and direct inhibition of lipid peroxidation [25,26]. Moreover, no changes in cholesterol were found, probably because diosgenin inhibits cholesterol absorption [27] and regulates its metabolism. Flavonoids present a great diversity of biological activities, such as antioxidant effects or modulation of enzymatic activity among others [28]. Among the flavonoids previously identified in *guishe* crude extracts, Morreeuw et al., 2021 [13,14,29], reported the aglycon flavonols (quercetin, kaempferol, and myricetin), flavanone (hesperitin and naringenin), flavanols (catechin and epicatechin), anthocyanidins (cyanidin and delphinidin), and flavones (apigenin), which are known for their antioxidant and cardioprotective benefits [30]. In addition, the presence of methylated, sulfated, gallated, and glycosylated flavonoids in *guishe* [13] and *A. lechuguilla* leaves [30] suggested high antioxidant and immunomodulatory effects of the extracts. All of these phytochemicals were found in the hydrolyzed extracts, and, in particular, anthocyanin and flavonol abundances were enhanced by applying the same hydrolysis condition as was used to prepare the tested extracts in this study [29]. Thus, the observed effects on lipid metabolism and antioxidant homeostasis of DM treatment could be attributed to the synergetic effect of the phytochemicals—saponins and flavonoids—described in the *guishe*. Further analysis using purified fractions could be helpful to elucidate their individual effects on DM treatment. 

## 3. Materials and Methods

### 3.1. Animals and Diabetes Induction

All experiments were conducted following the recommendations of the Mexican Federal Regulations for the Use and Care of Animals (NOM-062-ZOO-1999, Ministry of Agriculture, Mexico) [31] and approved by the institutional Ethics Committee of CIBNOR (March 10, 2017). Male Wistar rats weighing 150–240 g were provided by the IIQB-UMSNH animal house. Rats were fed and drank tap water ad libitum. Animals were kept under standard laboratory conditions (12 h light–dark cycles, 25 °C, and 80% humidity). Animals were set into eight groups as follows: control + vehicle; control + 150 mg/kg of extract; control + 300 mg/kg of extract; control + 600 mg/kg of extract; diabetic + vehicle; diabetic + 150 mg/kg of extract; diabetic + 300 mg/kg of extract; diabetic + 600 mg/kg of extract. To induce experimental DM, 60 mg/kg of fresh STZ (Sigma, St. Louis, MO, USA) was injected intraperitoneally [32]. Control animals received citrate buffer (pH 4.5). Three days later, DM was confirmed by measuring blood glucose levels using an Accutrend^®^ Plus System (Roche™, Manheim, Germany); rats with glucose levels greater than 200 mg/dL were considered for the protocols. After DM had been established, treatments were started, and glucose levels, cholesterol, and triglycerides were measured (overnight fasted) in all animals over a period of 5 weeks by sampling tail blood every 5 days until the end of the treatments.

### 3.2. Plant Material and Extract Preparation

*Agave lechuguilla* Torrey (Asparagaceae) was collected in Ejido Cosme, Ramos Arizpe, Coahuila, Mexico (25°52′03.6″ N; 101°11′0.96″ W) by regional and registered producers according to the NOM-008-SEMARNAT-1996 [33]. Lyophilizates from the *Agave lechuguilla* by-product *guishe* were obtained and provided by Reyes AG from CIBNOR S.C. La Paz, Baja California Sur, Mexico and employed in this study.

Extracts were prepared by macerating at 37 °C, continuously stirring for 2.5 h, and adding 10 mL of phosphate buffer (0.1 M, pH 4.0) per gram of dried-powdered plant material; an enzymatic mix (3.4 µL/g of plant) was also added (Ultraflo^®^ Max Novozymes A/S, Copenhagen, Denmark). After that, the solution was filtered and stored at 4 °C until use. The yield amount of extract was 476.5 mg/g of dried plant (47.65%).

### 3.3. Blood Chemistry

Blood was collected in non-heparinized red-top Vacutainer tubes (Becton Dickinson Div, Franklin Lakes, NJ, USA). Serum samples were obtained by centrifugation at 4000 rpm for 10 min (International clinical centrifuge, International Equipment, Woonsocket, RI, USA) and analyzed in a Cobas C111 analyzer by Roche^®^ for glucose, urea, creatinine, uric acid, total cholesterol, triglycerides, T bilirubin, D bilirubin, and alanine aminotransferase (ALT).

### 3.4. Kidney Mitochondria Isolation

Rats were euthanized by decapitation; kidneys were dissected immediately and gently homogenized in ice-cold medium containing 70 mM sucrose, 20 mM mannitol, 2 mM MOPS, and 1 mM EDTA (pH 7.4). Homogenate aliquots were stored at −80 °C to determine catalase activity. Mitochondria were isolated by a modified standard differential centrifugation, as described previously by Saavedra-Molina and Devlin, 1997 [34]. Mitochondria were purified in a Percoll gradient at 19,300 rpm (Beckman J2-MC, Life Sciences Division, Sacramento, CA, USA ); the mitochondrial pellet was suspended in ice-cold medium containing 220 mM mannitol, 70 mM sucrose, and 10 mM MOPS (pH 7.4). Aliquots were washed with phosphate buffer (pH 7.4) to determine lipid peroxidation. Mitochondria were stored at −80 °C until use. The mitochondrial protein content was determined with the Biuret test [35] using bovine serum albumin (BSA) as standard.

### 3.5. Lipid Peroxidation in Rat Kidney Mitochondria

Lipid peroxidation was performed according to Buege and Aust, 1978 [36], with slight modifications. In short, 1.0 mg/mL of mitochondrial protein was resuspended in a 0.1 M phosphate buffer (pH 7.4) and 3% BHT (butylated hydroxytoluene) and then mixed with a solution containing 0.375% thiobarbituric acid (TBA), 15% trichloroacetic acid, and 0.25 M HCl. The reaction was maintained for ~40 min in a boiling water bath. After that, tubes were cooled to room temperature and centrifuged at 7500 rpm for 5 min. The supernatant was measured at 532 nm in a Perkin Elmer Lambda 18 UV VIS spectrophotometer (Perkin Elmer Inc., Shelton, CT, USA). Lipid peroxidation levels were calculated based on the reaction of malondialdehyde (MDA) and TBA, using the molar extinction coefficient for MDA, 156 mM^−1^ cm^−1^.

### 3.6. Catalase Activity

Oxygen (O_2_) formation by catalase (CAT, EC 1.11.1.6) activity from kidney homogenates was determined from the dissociation of external H_2_O_2_ by using a Clark-type oxygen electrode [37] connected to a monitor (YSI 5300 Biological Oxygen Monitor, Yellow Springs, OH, USA). Previously, homogenates were centrifuged, and protein concentration from supernatants was determined. Briefly, 0.5 mg of protein resuspended in 0.1 mM phosphate buffer with 5 mM EDTA (pH 7.6) was introduced into the electrode chamber, maintained at 25 °C, and moderately stirred. The electrode was placed in position, and the basal reading was recorded for 30 s. After that, fresh H_2_O_2_ (6 mM) was added, and the oxygen pressure was continuously recorded for a further 120 s. Finally, 1.0 mM sodium azide was added, and recording was stopped 60 s later. To determine the catalytic activity, experiments with bovine catalase as standard were carried out in the same conditions. Results were expressed in units per milligram of protein (U mg^−1^ protein).

### 3.7. Mitochondria SOD Activity

Kidney mitochondrial superoxide dismutase (MnSOD) (EC1.15.1.1) activity was measured spectrophotometrically using the method described previously by Suzuki, 2000 [38], which uses xanthine/xanthine oxidase as an O_2_^•−^ generator, and nitro-blue tetrazolium (NBT) as a detector. Briefly, samples were placed into a plastic cuvette with a work solution (50 mM sodium carbonate, 0.1 mM xanthine, 0.025 mM NBT, 0.1 mM EDTA), xanthine oxidase (0.1 μM mL^−1^ in 2 M ammonium sulfate), and the sample or a blank. Changes in absorbance at 560 nm over 5 min were recorded in a Perkin Elmer Lambda 18 UV VIS spectrophotometer (Perkin Elmer, Inc., Shelton, CT, USA). Enzymatic activity was expressed in units per milligram of protein (U mg^−1^ protein). One unit of SOD activity is defined as the amount of enzyme needed to inhibit the reaction of O_2_^•−^ with NBT by 50%.

### 3.8. Determination of Glutathione (GSSG/GSH, GSSG, and GSH)

Total glutathione was determined according to Huerta-Cervantes et al., 2020 [39], with slight modifications. Briefly, 0.6 mg of mitochondrial protein was suspended in 0.1% Triton-X and 0.6% sulfosalicylic acid in a 0.1 M phosphate buffer with 5 mM ethylenediaminetetraacetic acid (EDTA), pH 7.5. The suspension was sonicated (Branson Ultrasonics™ S-450A Model Sonifier™ Analog Cell Disrupter, Brookfield, CT, USA) for 3 cycles on ice for 10 s each, followed by 2 freeze/defrost cycles. Finally, the samples were centrifuged at 6500× *g*, and the supernatant was placed in phosphate buffer with 100 µM 5,5′-dithiobis(2-nitrobenzoic acid) (DTNB) and 0.1 unit/mL glutathione reductase (GR) and incubated for 30 s. The reaction was started by adding 50 µM β-NADPH and monitored for 5 min at 412 nm using the kinetic mode on a Perkin Elmer Lambda 18 UV VIS spectrophotometer (Perkin Elmer, Inc., Shelton, CT, USA). Oxidized glutathione (GSSG) was obtained after reduced glutathione (GSH) derivate by incubating with 0.2% 4-vinylpyridine for 1 h at room temperature. GSH was calculated by subtracting GSSG from the total glutathione.

### 3.9. Determination of Glutathione Peroxidase (GSH-Px)

GSH-Px activity was determined according to Lawrence and Burk, 1976 [40], with slight modifications. Mitochondria (0.2 mg) were resuspended in a potassium phosphate buffer (50 mM, pH 7.4) plus EDTA (5 mM) and mixed with GSH (1 mM), NaN_3_ (1 mM), BSA (0.1 mg), and GSH reductase (100 mU/mL) and incubated for 5 min. Then, NADPH (100 µM) was added and incubated for 1 min further. Fluorescence was followed for 1 min at 352/464 nm excitation/emission wavelengths, and H_2_O_2_ (250 µM) was added. Changes in fluorescence were monitored over 3 min at 30 °C in a spectrofluorophotometer (Shimadzu RF-5301PC, Kioto, Japan). A blank reaction was carried out replacing the sample with deionized water and subtracted on each assay.

### 3.10. Statistical Analysis

The results were expressed as the mean ± SEM of at least three independent experiments. Statistical significance (*p* ≤ 0.05) was determined with Student’s *t*-test using Prisma 8.0. Analysis of variance (ANOVA) with post hoc Tukey HSD test was performed in every case for unbalanced data.

## 4. Conclusions

In conclusion, we found that *guishe* at doses of 300 mg/kg of body weight has a beneficial effect and can be used to treat abnormalities in serum lipids and to mitigate the oxidative stress occurring during diabetes. Nevertheless, further studies should be undertaken to determine the mechanism that regulates the serum triglyceride levels. Furthermore, it is important to mention that the biotechnological potential of *guishe* from *Agave lechuguilla* in the field of pharmacology is real. However, efforts need to be directed towards the establishment of purification and concentration strategies for some specific biomolecules.

## Figures and Tables

**Table 1:** Blood chemistry from rats treated with Agave lechuguilla–*guishe* aqueous extract.

Parameter	Units	Control	DM	Control 150 mg/kg	DM 150 mg/kg	Control 300 mg/kg	DM 300 mg/kg	Control 600 mg/kg	DM 600 mg/kg
Glucose	mg/dL	131.67 ± 1.20	374.67 ± 38.68 *	118.00 ± 4.04	445.67 ± 21.54 *	116.00 ± 2.08	386.67 ± 22.82 *	134.67 ± 0.33	344.67 ± 22.45 *
Urea	mg/dL	37.70 ± 0.61	80.59 ± 3.73 *	37.43 ± 1.50	145.48 ± 1.28 *	144.51 ± 1.19	222.97 ± 13.47 *	228.91 ± 5.81	322.38 ± 8.48 *
Creatinine	mg/dL	0.27 ± 0.03	0.30 ± 0.00	1.13 ± 0.09 *	0.63 ± 0.03	1.23 ± 0.09	1.07 ± 0.17	1.27 ± 0.12	1.07 ± 0.03
Cholesterol	mg/dL	150.67 ± 0.33	152.67 ± 1.20	151.00 ± 0.58	155.67 ± 0.67	154.00 ± 2.31	154.67 ± 1.86	155.00 ± 2.65	151.67 ± 0.67
Triglycerides	mg/dL	144.33 ± 1.86	126.33 ± 1.20 *	99.67 ± 0.33	117.00 ± 2.00*	101.00 ± 2.89	85.00 ± 2.31 *	90.00 ± 3.21	86.00 ± 4.93
Uric Acid	mg/dL	1.03 ± 0.15	1.97 ± 0.13 *	3.13 ± 0.15	2.77 ± 0.32	3.30 ± 0.21	4.30 ± 0.25 *	3.70 ± 0.06	2.80 ± 0.00
T Bilirubin	mg/dL	0.10 ± 0.00	0.13 ± 0.03	0.17 ± 0.03	0.17 ± 0.03	0.23 ± 0.07	0.23 ± 0.09	0.17 ± 0.03	0.20 ± 0.06
D Bilirubin	mg/dL	0.07 ± 0.03	0.10 ± 0.00	0.03 ± 0.03	0.03 ± 0.03	0.07 ± 0.03	0.03 ± 0.03	0.03 ± 0.03	0.07 ± 0.03
ALT	U/L	106.9 ± 11.15	127.60 ± 7.39	190.60 ± 9.37	380.77 ± 11.34 *	215.20 ± 0.85	573.80 ± 20.71 *	195.40 ± 15.59	644.03 ± 4.29 *

Values express the mean ± SEM of at least *n* = 3 rats. ANOVA with post hoc Tukey HSD test for unbalanced data was performed. * *p* ≤ 0.05 controls versus DM groups. Abbreviations: T bilirubin, total bilirubin; D bilirubin, direct bilirubin; ALT, alanine aminotransferase. All determinations were carried out in blood serum obtained at the end of the 5 weeks of treatment with an aqueous extract from Agave lechuguilla–*guishe*. Source: authors’ own elaboration.

**Table 2:** Enzymatic determination for superoxide dismutase, catalase, and glutathione peroxidase.

Enzyme	Units	Control	DM	Control 150 mg/kg	DM 150 mg/kg	Control 300 mg/kg	DM 300 mg/kg	Control 600 mg/kg	DM 600 mg/kg
mSOD	U mg^−1^ prot	178.9 ± 17.7	321.9 ± 5.9 *	244.2 ± 5.2	313.7 ± 12.4 *	265.0 ± 14.8	303.2 ± 27.0	216.5 ± 3.3	193.8 ±17.8
Catalase	U mg^−1^ prot	31.2 ± 1.6	34.7 ± 0.2	22.6 ± 0.5	21.6 ± 1.0	27.5 ± 1.9	24.1 ± 1.7	36.6 ± 0.9	20.1 ± 0.6 *
GSH-Px	µM Min^−1^ Mg^−1^ prot	33.4 ± 0.1	29.8 ± 0.4 *	42.5 ± 0.5	37.7 ± 1.3 *	95.2 ± 0.8	94.4 ± 1.0	98.3 ± 1.6	94.5 ± 1.2

Values express the mean ± SEM of at least *n* = 3 rats. ANOVA with post hoc Tukey HSD test for unbalanced data was performed. * *p* ≤ 0.05 controls versus DM groups. Abbreviations: mSOD, mitochondrial superoxide dismutase; GSH-Px, glutathione peroxidase. Catalase was determined in kidney homogenates, and mSOD and GSH-Px were determined in isolated kidney mitochondria—in both cases, at the end of the 5 weeks of treatments. Source: authors’ own elaboration.

## Data Availability

The data presented in this study are available on request from the corresponding author.
